# Post traumatic type 1 diabetes mellitus (insulin-dependent): a case report

**DOI:** 10.11604/pamj.2014.19.328.5632

**Published:** 2014-11-27

**Authors:** Rabie Karrouri

**Affiliations:** 1 Psychiatrist, Department of Psychiatry, Hopital Militaire Moulay Ismail, Meknès 50000, Morocco

**Keywords:** Trauma, diabetes, stress, pathogenesis, children

## Abstract

Most researchers have studied the influence of life stress as precipitating the onset of type 1 diabetes, but as the relationship between severe psychological trauma and diabetes has been a rarely studied subject in paediatric age group. Here, we report the case of a 10-year-old Libyan boy, without personal or familial diabetes mellitus history, which is presented to Moroccan medico-surgical field hospital, installed in Tunisia for refugees of the Libyan revolution, for type 1 diabetes appeared immediately after severe psychological trauma.

## Introduction

In Diagnostic and Statistical Manual of Mental Disorders, Fifth Edition, trauma is described as the experience of intense fear, helplessness, horror, or disorganized and agitated behaviour in response to exposure to an event -directly or as a witness- that caused or threatened serious injury or violation of body integrity [1]. Type1-diabetes (T1D) or insulin-dependent diabetes or juvenile-onset diabetes is a chronic autoimmune disease characterized by the destruction of pancreatic β-cells in the islets of Langerhans resulting in insulin deficiency and hyperglycaemia [2, 3]. T1D is one of the most serious forms of diabetes mellitus; it leads to a lifelong dependency on daily insulin injections. The pathogenesis of T1D is strongly linked to autoimmunity against the islet β-cells [4], but not necessarily its primary cause [2]. Many studies further support the opinion that environmental factors are also important [4], but the role of stress in the pathogenesis of T1D remains very controversial and confusing [1, 5]. The capacity of psychological trauma to produce T1D remains little studied, however no study can confirm or deny that stress events may facilitate the occurrence of this disease. In the present report, we present the case of a child who manifested a T1D just after severe psychological trauma.

## Patient and observation

A 10 years old Arab Libyan boy, seventh of 8 children-family of modest shepherds. While helping his older brothers to keep the sheep, a “Grad”-type shell has dropped near them. His brothers fled while he, stunned by the sudden event, did not move from his place. “I was scared”, he said with a smile on his lips, “I do not understand why!” Covered with sand and abandoned by his brothers, the little boy, remained so long; motionless; in the same position until his father, alerted by his brothers of his disappearance, picked him up. After this event, the family decided to leave the country to take refuge in Tunisia. The next day after the incident, the father noticed his son is frequently asking for a drink and going to the toilet to urinate. A few days went by; he began to quickly loose weight despite of eating almost all the time. One week after the event, the patient was brought by his father to a paediatric consultation in our medical and surgical field hospital. Fasting plasma glucose (FPG) was made the next day morning to 430 mg/dl (23.86 mmol/l) and haemoglobin A1C levels at the 14%. A family survey (since the whole family had taken refuge) revealed no familial cases of diabetes, nor similar cases of T1D. The diagnosis of T1D was retained; the boy was put on insulin-therapy, and then redirected to our tent for a psychiatric care.

During the psychiatric examination, the boy was very cooperative. He presented no difficulty in sleeping or a repetition syndrome (characteristic of post-traumatic stress), but rather a change in character reported by his father apparent in: Irritability, aggression and a constant search to dominate his peers and brothers. On top of that, and in contradiction with his usual character where he spent days in desert with his grandfather and his brothers keeping camels, it became difficult for him to leave home, and refused any hospitalization for assessment purpose, even short and accompanied. He had good school grades before the events but his level cannot be evaluated after because the refugees were in forced vacation. He bought a plastic gun to play with, “it is to kill Gaddafi” he said, aware of his disease, he continues “it was he who put sugar on my blood by his”jrad ”. The diagnosis of post-traumatic stress disorder (PTSD) was withheld and the patient received psychotherapy alone, especially because the chemotherapy prescription was refused by the family for cultural reasons (makes him look mad, frowned upon by the environment...).

## Discussion

While PTSD symptoms are common in older adolescent and adults, the cognitive and expressive language limitations of young children oblige therefore the clinician to attempt to deduce symptoms from behavioural observations of the child and from direct reports from parents, teachers and other observers in the child's milieu. The traumatized children often present an array of symptoms not typically assessed by existing scales [6]. These may include the loss of recently acquired developmental skills (regression), the emergence of new fears or re-activation of old ones, accidents and reckless behaviour, separation anxiety (often manifested in anxious clinging), and psychosomatic complaints (stomachaches, headaches...). Additionally, young children may express post-traumatic anxiety through hyperactivity, distractibility and increased impulsivity. This suggests that it might be more precise to make a diagnosis of PTSD among children based on the intensity of symptoms and their relationship to functional impairment, rather than on the threshold number of symptoms [6].

T1D is most often diagnosed in children and adolescents, usually presented with a classic trio of symptoms (i.e., polyuria, polydypsia, weight loss and sometimes with polyphagia) alongside with overt hyperglycaemia [2]. Fasting plasma glucose (FPG) ≥ 126 mg/dl (7.0 mmol/l), after an efficient fasting (no caloric intake for at least 8 hours), is a sufficient criteria to diagnose diabetes mellitus according to the American Diabetes Association [2]. A1C is a widely used marker of chronic glycaemia, reflecting average blood glucose levels over a 2 to 3 months period of time. The islet cell autoantibody test is not in clinical routine use for diagnosing T1D [7], especially before obvious clinical symptoms. The role of stress in the onset of diabetes mellitus, including those of T1D, has long been a topic of discussion, but only rare cases of T1D posttraumatic can be retained and on strict criteria of severity of traumatic event, precession between trauma and diabetes within a short period of time, and no previous symptoms of diabetes before trauma [8]. If studies of stress related diabetes seem to be many, it rather deal with the relationship of diabetes to minor injuries not involving life-threatening, like infections, prenatal difficulty, parents conflicts or hypoglycaemia [1, 3, 5]. While the impact of severe trauma show an increase in the incidence of diabetes mellitus due to severe traumatic events life-threatening [9], especially war events similar to ours [10]. A period of time up to 24 months is suggested by many authors [3] to retain a causal relationship between the traumatic events and the onset signs of diabetes mellitus. In our case, the installation of the triad of diabetes mellitus was in a very short time (just one week).

The involvement of stress in the pathogenesis of diabetes is fundamentally different depending on whether the installation causes the disease symptoms or would it simply precipitate the final stages. The known absence of diabetes before the traumatic event leads to a high probability of post-traumatic diabetes [8]. The absence of symptoms of diabetes before the traumatic event was clear from the interviews conducted with the entourage of our case. In fact, the family was alerted by the installation of unusual symptoms for them especially given the absence of family history of diabetes mellitus, which precipitated their consultation. The post-traumatic diabetes is insulin-dependent in most cases.

## Conclusion

A severe psychological traumatism may play a more etiological role in the pathogenesis of T1D in a non predisposed child. A better understanding of how the trauma is involved in the diabetogenesis will - probably - identify important mechanisms or stages to develop a curative or preventive treatment of T1D. The need for a systematic research on post-traumatic diabetes in children is to be noted.
